# Anxiety of microbially synthesized Fe_3_O_4_-SPIONs on embryonic/larval ontogeny in red tilapia (*Oreochromis* sp.)

**DOI:** 10.1007/s00253-024-13386-x

**Published:** 2025-01-07

**Authors:** Samia S. Abouelkheir, Mona M. Mourad

**Affiliations:** National Institute of Oceanography and Fisheries (NIOF), Cairo, Egypt

**Keywords:** Fe_3_O_4_-SPIONs, *Bacillus* sp., Cytotoxicity, *Oreochromis* sp., Malformation, Ontogeny

## Abstract

**Abstract:**

Iron oxide nanoparticles, recognized for their superparamagnetic properties, are promising for future healthcare therapies. However, their extensive use in medicine and electronics contributes to their discharge into our environments, highlighting the need for further research on their cellular damage effects on aquatic organisms. While the detrimental properties of other compounds have been stated in the early-life stages of fish, the cytotoxic consequences of superparamagnetic iron oxide nanoparticles (SPIONs) in these stages are still unexplored. Therefore, using the red tilapia (*Oreochromis* sp.) as a model organism, this study is the first to talk about the subtle cellular alterations caused by biologically induced biomineralized Fe_3_O_4_-SPIONs by *Bacillus* sp. in the early-life stages. Once the red tilapia eggs were fertilized, they were challenged to different doses of SPIONs (0, 5, 10, 15, and 30 mg/l), and their tenfold increases (50, 100, 150, and 300 mg/l) for 72 h. The hatching rate, malformation rate, body length, and deformities of the larvae were all studied. Our research showed that iron oxide nanoparticles were harmful to the early stages of life in red tilapia embryos and larvae. They slowed hatching delay, a decrease in survival rate, an increase in heart rate, bleeding, arrested development, and membrane damage and changed the axis’s physiological structure. Additionally, results indicated numerous deformities of red tilapia larvae, with lordosis, kyphosis, and scoliosis once subjected to 50 and 150 mg/l of SPIONs concentrations, respectively. This study could assist us in recognizing the risk and evaluating the disrupting potential of nanoparticles. The key objective of this inquiry is to describe the existing features of the produced magnetite SPIONs (29.44 g/l) including their morphological, chemical, and magnetic characteristics. Illustrate their current role in medicinal applications and aquatic organisms by studying in vivo cytotoxic effects to motivate the development of enhanced SPIONs systems. As a recommendation, more research is needed to completely understand how various exposure endpoints of SPIONs disturb the bodies of red tilapia in the early stages.

**Key points:**

• *Biogenic SPIONs: a material of the future.*

• *Characterization is essential to assess the functional properties of the produced SPIONs.*

• *Fe*_*3*_*O*_*4*_*-SPIONs’ impact on the red tilapia ontogeny.*

**Graphical abstract:**

**Figure:**
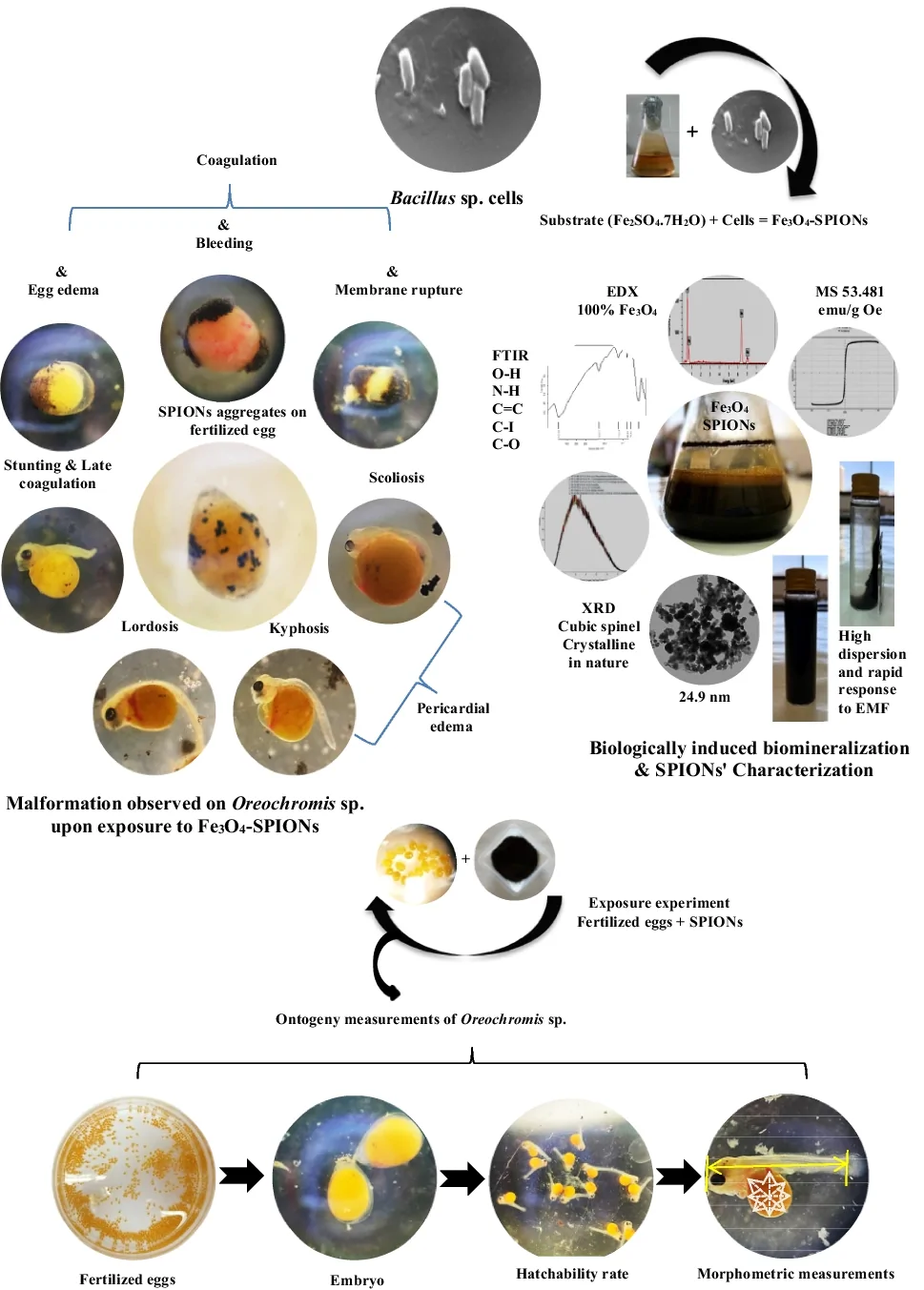

## Introduction

Nanotechnology focuses on particles fluctuating in dimensions from 1 to 100 nm, including magnetic nanoparticles (MNPs) with cores of Fe, Co, Ni, and Al (Gatoo et al. 2013; Ng et al. 2015; De Oliveira et al. 2017). Superparamagnetic iron oxide nanoparticles (SPIONs), mainly magnetite (Fe_3_O_4_), maghemite (γ-Fe_2_O_3_), and ferrites with dimensions beneath 100 nm and superparamagnetic traits, are frequently crystalline (Chen et al. 2010). An external magnetic field may govern these ferric and ferromagnetic nanoparticles, exhibiting magnetism in one particular domain at the Curie temperature value (Justin et al. 2017). The exceptional manners of SPIONs, during an exterior magnetic field, reveal their magnetism as a single domain, attracting research for directed manipulation and straightforward removal of employed SPIONs (Samrot et al. 2012). Agglomeration is a significant issue in nanoparticles, necessitating strategies like coating them with biopolymers or other biocompatible chemicals (Ashby et al. 2014; Faraji et al. 2010). Nanoparticle modification promotes monodispersion and facilitates linking them with further molecules. SPIONs’ superior surface-to-volume proportion makes them rapidly oxidized; forming accumulates in fluid systems (Wu et al. 2015a). Modifying their surface improves their capabilities for various purposes (Mohammad et al. 2010; Malhotra et al. 2020).

SPIONs are broadly employed in therapeutic procedures (Ling et al. 2015), among which are the use of MRI (magnetic resonance imaging), MPI (magnetic particle imaging), separation of biomolecules, directed medicines besides gene delivery, and MFH (magnetic fluid hyperthermia) that is a way to raise the temperature surrounding malignant cells (Mahmoudi et al. 2011b; Dadfar et al. 2019). For instance, significant breakthroughs were achieved to connect SPIONs with nanoparticles of gold, allowing for more effective hyperthermia therapy of cancer (Mohammad et al. 2010). SPIONs’ magnetic characteristics, as well as their flexibility to be manufactured in an array of dimensions and forms, contribute to their vast range of applications. SPIONs are recommended to display considerable magnetism, a size of less than 100 nm, and a narrow range of sizes to be applied in all of the aforementioned applications (Lee et al. 2015; Wu et al. 2015b; Dadfar et al. 2020).

The growing manufacturing and consumption of iron oxide nanoparticles will eventually increase vulnerability among individuals and the biosphere. Iron oxide nanoparticles are being employed for a growing number of purposes due to their superparamagnetic traits, involving cell labeling, separation, tracking, cancer therapy, and diagnostic imaging (Heo et al. 2014; Malhotra et al. 2020). They are particularly used for image-guided drug delivery and tissue engineering, combining diagnosis and therapy. This broad applicability indicates the incorporation of numerous iron oxide nanoparticle-based products in subsequent medical therapies (Dadfar et al. 2019). Flame spray pyrolysis (FSP), for example, is a bottom-up nanomanufacturing technique that allows continuous production of metal oxide nanoparticles with high yields and low costs at significant rates in easily reproducible single-step procedure on a large scale (up to 10 kg/h) for pharmaceutical and biotechnological applications (Estévez et al. 2023). Chaudhari et al. (2024) also provided descriptions of sustainable iron oxide nanoparticle productions and their roles in remediating the environment. So it is vital to explore the possible medical and ecological consequences of iron oxide nanoparticles on human beings, non-human life forms, and ecological systems (Zhu et al. 2012; Hajiyeva et al. 2023). Still, it appears that there is insufficient information regarding the risks associated with these nanosized substances to biological functions (detrimental communications inside the body, deposition in tissues, and discharge into the body), considering their behavior and mechanism of interaction are entirely unexplored due to their distinctive features (Oliveira-Filho et al. 2016; Malhotra et al. 2019). Overall, they remain intact along with colloidal forms that may either stay suspended or combined. Their structure and form contribute to our knowledge of their attitudes and destiny in their surroundings, as well as how they will communicate with living beings.

The majority of investigations on the probable influence or hazard of iron oxide nanoparticles have been carried out on animals (such as mice and rats) and/or numerous cell lines (Mahmoudi et al. 2011a). Magnetic nanoparticles have been extensively researched in aquatic toxicity, with harmful effects observed in fish gills, the digestive system, the liver, and the brain (García et al. 2011; Oliveira-Filho et al. 2016). By producing reactive radicals and interfering with normal physiological processes, the endocytosis process, which engulfs MNPs, may induce cell toxicity (Malhotra et al. 2019). Consider how released NPs may transfer via a food chain to algae, zooplankton, aquatic invertebrates, and fish to comprehend toxicity in aquatic creatures (Rana and Kalaichelvan 2013). Consequently, research on the ecological toxicity of nanoparticles to aquatic creatures is crucial (Cerrillo et al. 2017). To determine how particular magnetic nanoparticles behave inside cells and the cell system throughout the body, it is crucial to understand their safety standards for environmental exposure. The probability of these connections causing significant cell damage relies on the exact parameters adopted for the construction of a nanoparticle (Gajewicz et al. 2012; Wehmas et al. 2015).

The fabrication, biological activity, and potential utilization of an array of nanoparticles including metal complexes and their free forms have all been the subject of recent investigations (Hajiyeva et al. 2024). Research has shown that MNPs having a variety of surface coatings are hazardous. For instance, dextran-coated Fe_3_O_4_ inhibited cell growth and induced cellular death in the same way that naked iron oxide particles did at 50 g/ml (Malhotra et al. 2019). In other research, degradation of lipids, metal ion discharge, cell wall deterioration, and the emergence of reactive oxygen species (ROS) have been stated next MNP contact in vitro and in vivo (Kovrižnych et al. 2013; Ruiz et al. 2015; Ahamed et al. 2016; Bisht et al. 2016; De Oliveira et al. 2017; Zheng et al. 2018). Di-mercapto-succinic acid (DMSA)-coated Fe_3_O_4_ MNPs were administered to rats and aggregated in their spleens, livers, and lungs with minimal harm to the animals (Ruiz et al. 2015). Starch-coated MNPs also caused big changes in the genes that were expressed in different ways in the liver and gill transcriptome of adult zebrafish (Zheng et al. 2018).

The research additionally provides insight into how these nanoparticles bioaccumulate in nature, particularly in fish, mollusks, protozoa, and helminths, among other aquatic creatures (Hasanova et al. 2021; Rzayev et al. 2022; Nasirov et al. 2024). Moreover, once aquatic beings are subjected to nearby toxins at sensitive phases, they reply rapidly. Hence, they provide a useful paradigm for understanding toxicity. To this purpose, assessing MNP toxicity in adult zebrafish gives valuable data for particular improvements in healthcare and environmental policies. Therefore, researchers are currently conducting experiments on model species like rats, mice, and zebrafish (Malhotra et al. 2019), as in vivo investigations provide more robust and reliable data for toxicity measures than in vitro research.

Tilapia is an important species of cultivated fish, second only to carp. They are suitable for farming owing to their tolerance to insufficient conditions, rapid development, simplicity of reproduction, and the tendency to turn organic and household waste into superior protein. Tilapias are also referred to as aquatic chickens because of their texture and flavor. They are appropriate for large-scale farming systems in developing countries and are suggested as a possibility for small farmers in social development projects (Zuniga-Jara and Goycolea-Homann 2014). Red tilapia is a hybrid tilapia that evolved from the blue tilapia (*Oreochromis aureus*) and the Mozambique tilapia (*Oreochromis mossambicus*) (Mourad et al. 2022). Luo et al. (2023) utilized red tilapia as an aquaculture model due to its adaptability, productivity, and nutritional value. Red tilapia is salt-tolerant and omnivorous and inhabits various water bodies. They migrate to the top layer for foraging as the water gets warmer. Their bright, appealing body and tasty meat make them a popular choice in aquaculture. Nor et al. (2020) found red hybridized tilapia to be an appropriate candidate for studying fish infections and immunizations for viral infection due to their fast-growing, cheap, and disease-resistant nature. Also, Huang et al. (2021) employed red tilapia as an experimental fish to study the interaction between older and virgin microplastics.

Furthermore, larval malformations have been utilized as an indicator for contamination of water since the earliest stages of development are susceptible to contaminants being exposed, which can have a harmful impact on subsequent developmental processes. For example, Nile tilapia embryos and larvae that were subjected to waste from palm oil mills got birth defects like pericardial edema, lordosis, kyphosis, and a curved tail (Muliari et al. 2020). To the greatest extent of our information, no studies were conducted regarding the cytotoxicity of SPIONs in the infancy phases of red tilapia. Therefore, the goal of this investigation was to look into how biosynthesized magnetite (Fe_3_O_4_) nanoparticles affect the rates of survival, hatchability, and embryonic development in the initial phases of this fish species’ life.

## Materials and methods

### Marine bacterium strain

The marine *Bacillus* sp. strain with accession number GU191141 used throughout this study was isolated by Samia Saad Abouelkheir (Abouelkheir et al. 2018) and deposited in the National Research Centre culture collection EMCCN-NRC under number 3081.

### Microbiological medium used for bacterium cultivation

Nutrient broth (NB) medium was used for seed culture preparation and cultivation in shake flasks (Majeed et al. 2020). This medium was composed of lab-lemco powder, 1; yeast extract, 2; peptone, 5; and sodium chloride, 5 in g/l seawater, pH 7.0.

### Biosynthesis of Fe_3_O_4_-SPIONs

Iron oxide nanoparticles were biologically synthesized using *Bacillus* sp. The bacterial cells were allowed to grow in a 250-ml Erlenmeyer flask containing 100 ml of NB medium. The synthesized particles were obtained after mixing 0.1 g of wet bacterial cells with 100 ml of 50 mM FeSO_4_.7H_2_O in a 250-ml Erlenmeyer flask at regular stirring on a rotary shaker (120 rpm) at 30 °C and pH 9 for 24 h. The produced particles were visually detected by the color alteration to black (Abouelkheir et al. 2023).

### Fe_3_O_4_-SPIONs characterization analyses

The prepared nanoparticles were characterized using X-ray diffraction (XRD) (Bruker XRD-D2 Phaser) (Bruker, Karlsruhe, Germany) to determine the crystal phase structure using a copper X-ray source working at 30 kV and 10 mA. Scans were recorded at 2°/min in the 2θ range of 10–100° (Central Laboratory, Faculty of Science, Alexandria University). The crystallite size (CrS) was assessed via Scherrer’s equation (Abouelkheir et al. 2020):

1 $$CrS=K\lambda /\left(\beta \mathrm{cos}\theta \right)$$

where (*k*) is the dimensionless Scherrer constant = 0.94, (λ) is the X-ray wavelength = 1.54184 nm, (β) is the peak full width at half maximum in radians, and (θ) is the diffraction angle in radians. Fourier transform infrared spectroscopy (FTIR) (Bruker Tensor 37, Billerica, Massachusetts, USA) analysis was done to define the spatial distribution of the functional groups. The transmission electron microscope (TEM) (Jeol. 1400Plus, Tokyo, Japan) allows studying the surface morphology and size distribution. The purity of the biomanufactured particles was examined by energy dispersive X-ray (EDX) investigation by a scanning electron microscopy (SEM) (Jeol JSM-IT 200, Tokyo, Japan) equipped with an EDX unit with an acceleration voltage of 20.00 kV and a magnification of 500. A magnetic property was assessed by the Lakeshore 7410 vibrating sample magnetometer (VSM) (NanoMagnetics Instruments, Summertown, Oxford, UK) (Abouelkheir et al. 2023). Electromagnetic movement assay is utilized to determine the value of zeta-potential (ζ-potential) via dynamic light scattering (DLS) using Zetasizer ver.8.02 (Malvern Panalytical Ltd., London, UK) (Estévez et al. 2023).

### Preparation of different Fe_3_O_4_-SPION solutions

A powder form of ultra-fine Fe_3_O_4_-SPIONs was used to form various concentrations. The concentration gradients of nFe_3_O_4_ studied in this investigation were 5, 10, 15, 30, 50, 100, 150, and 300 mg/l with water as a control. These solutions were prepared by dispersing MIONP in sterilized brackish water (15 ppm). The mixture was then shaken continuously with magnetic stirring every day before dosing to keep the NPs from settling. The rate of dispersion was excellent at the final working concentration, with a few agglomerates of NPs being noticeable in prepared solutions (Zhu et al. 2012, 2017).

### Broodstock and egg maintenance

**Fig. 1:**
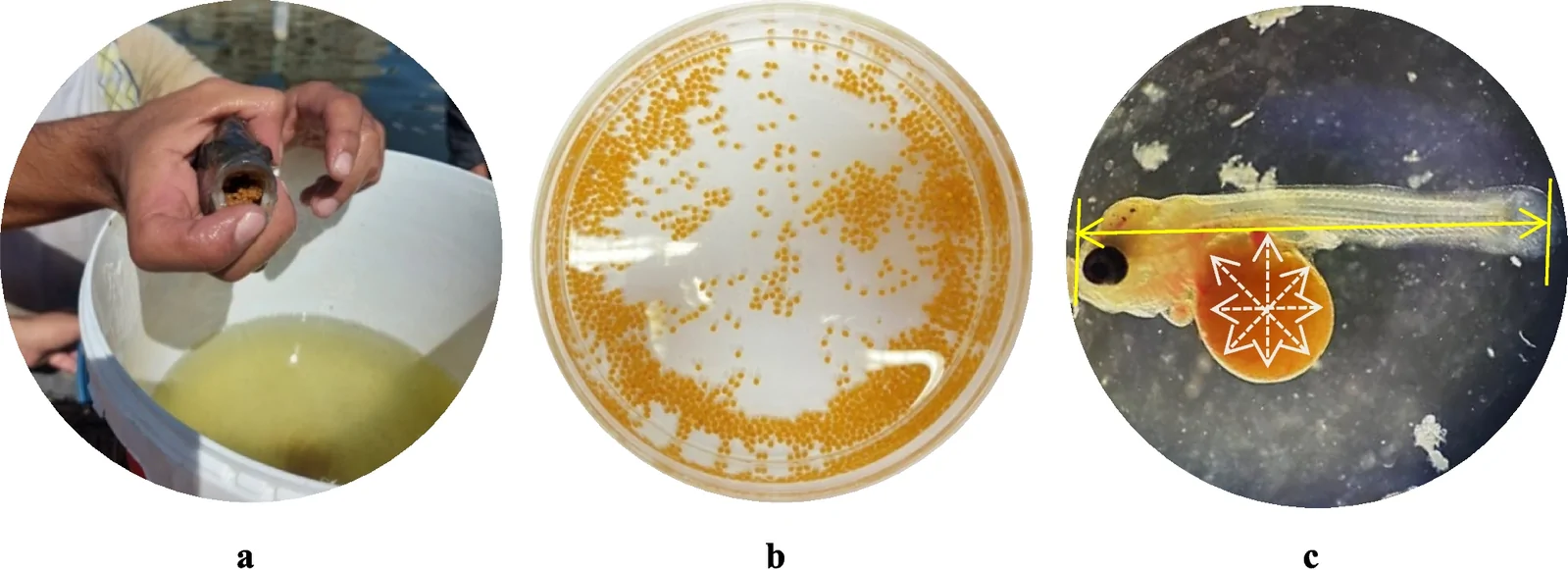
Mouthbrooder red tilapia *Orechromis* spp. carries newly fertilized eggs into the mouth cavity (**a**); morphometric measurements of laboratory fertilized eggs (**b**) spawned to larvae (**c**)

Red tilapia eggs (*O. aureus* × *O. mossambicus*) were gathered from naturally spawning broodstock that weighed about 120 ± 10 g and had one male for every three females (Muntaziana et al. 2011). At the late gastrula and pro-organogenesis stages, fertilized eggs were taken from the broodstock mouth to transport jars and washed with fresh water. Eggs that were not fertilized, not asymmetrical, contained vesicles, or damaged were thrown away. When the eggs got there, they were put in a semi-static glass aquarium system with purified seawater that had been diluted with de-chlorinated tap water. This was done until the salinity reached 15 ppt and the pH reached 7. At room temperature, oxygen saturation (dissolved oxygen, DO = 6.87 ± 0.06 mg/l) was kept up by continued agitation. To prevent metabolic byproduct contaminants, aquarium whole water was changed every 24 h (Fig. 1a, b).

### Experimental exposure of fertilized eggs to Fe_3_O_4_-SPIONs (Zhu et al. 2012)

One day after fertilization, healthy, viable eggs were transferred to a 9-cm plate at a rate of 20 eggs per 10 ml of Fe_3_O_4_-SPIONs. This was implemented in triplicate for each treatment. The embryo cytotoxicity test design was followed according to the Organization for Economic Co-operation and Development (OECD 212) guidelines (Braunbeck and Lammer 2006). The embryo culture medium, including the Fe_3_O_4_-SPIONs test solution, was prepared immediately prior to the exposure. Tens of milliliters of Fe_3_O_4_-SPIONs test solution with concentrations of 5, 10, 15, and 30 mg/l, as well as their tenfold increase of 50, 100, 150, and 300 mg/l, were added to each labeled plate and looked at for 24, 48, and 72 h post-fertilization. A control plate was prepared with the culture medium free from the Fe_3_O_4_-SPIONs. The plates were coated with transparent plastic sheets to avoid solution vaporization, which provided aeration via fixed pores on the surface. A Delta LabTech shaker (model LSI-3016A, Bangkok, Korea) was used to keep the plates with embryos at a constant speed, temperature, and light/dark cycle (150 rpm, 28 °C ± 2, and 10 h/14 h). Daily dead embryos and larvae were discarded at the end of each day with culture medium and Fe_3_O_4_-SPIONs dose replacement and recorded. As part of the daily culture media exchange procedure, the old medium was removed, and embryos were rinsed with blank water (15 ppt) before adding a new medium. Both authors promptly incorporated this procedure to decrease the stress limit to a minimum.

### Developmental toxicity and morphometric parameter measurement

Throughout the duration of the exposure period, the developmental status of the red tilapia embryos and larvae was documented at specific intervals (day 1, day 2, and day 3) using a stereomicroscope (Olympus SZ61, model SZ2-LGB, Tokyo, Japan). The evaluation was done as described previously by Fujimura and Okada (2007). The developmental toxicity was determined by measuring the parameters of embryo/larva egg retardation, hatching, survival, and malformation rate, as well as the standard length (SL) and diameter of the yolk sac. The hatchability rate was assessed via the following equation:

2 $$Hatchability\;\%=\left(No.\;of\;hatched\;eggs/No.\;of\;fertile\;eggs\right)\times100$$

The samples were digitally photographed at various magnifications using an iPhone 11 (model number MWM52AH/A, resolution 4032 × 3024, USA). The images were analyzed using the Image J 1.48v program (NIH Image, USA), and the standard length (SL) and diameter of the yolk sac in four dimensions (presented as minimum and maximum) were determined for all organisms (Fig. 1c). The qualitative measurements of teratogenicity and morphological effects of exposure to various concentrations of Fe_3_O_4_-SPIONs were described according to von Hellfeld et al. (2020) and Jezierska et al. (2000). Malformations were identified and documented daily in both the treatment and control groups of the embryos and larvae, including ones from deceased organisms.

### Statistical analysis

All trials were done in triplicate. A statistical estimation of the data as the mean was done, presented, and calculated by a polynomial regression, order 2, using Past3 software (PALSTAT, Ireland) and Origin Pro 8.1 (OriginLab, Northampton, Massachusetts, USA). Each embryo/larval group’s standard deviation (SD) and mean of the data were individually compared to the control to detect significant differences. A one-way analysis of variance (ANOVA) using SPSS version 20 (SPSS, Richmond, VA, USA) was conducted for the embryo/larval bioassays (egg retardation, hatchability, survival and malformation rates (%), yolk sac diameter, and stander length) in accordance with Dytham’s (2011) guidelines. LSD and Duncan’s multiple range tests were used as indicators for significant variations in the means between the groups. A statistically important result was considered as *P* < 0.05.

## Results

### Bacterial synthesis of Fe_3_O_4_-SPIONs

**Fig. 2:**
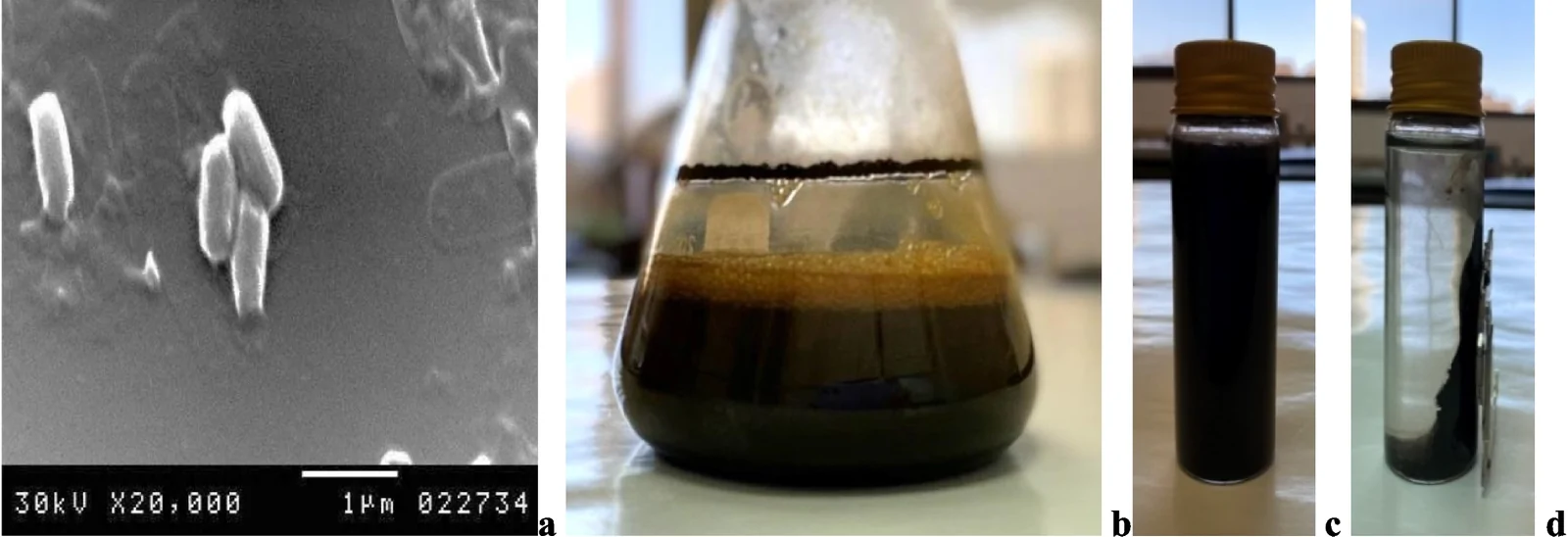
SEM image showing *Bacillus* sp. cells (**a**), biologically induced biomineralization of Fe_3_O_4_-SPIONs (**b**), highly dispersed (HD) Fe_3_O_4_-SPIONs in solution (**c**), and rapid response to the external magnetic field (RREMF) (**d**)

Biologically synthesized SPIONs were successfully produced by *Bacillus* sp. cells (Fig. 2a) through extracellularly induced biomineralization activity. The collected cells of the strain, about 1 g, were challenged with 50 mM FeSO_4_.7H_2_O which produces a dark brown to black colloid around 29.44 g/l dry weight which approves the development of iron oxide nanoparticles (IONPs) (Fig. 2b). The produced solution was highly dispersed (HD) (Fig. 2c) and showed a rapid response to an external magnetic field (RREMF) (Fig. 2d).

### Characterization of Fe_3_O_4_-SPIONs

**Fig. 3:**
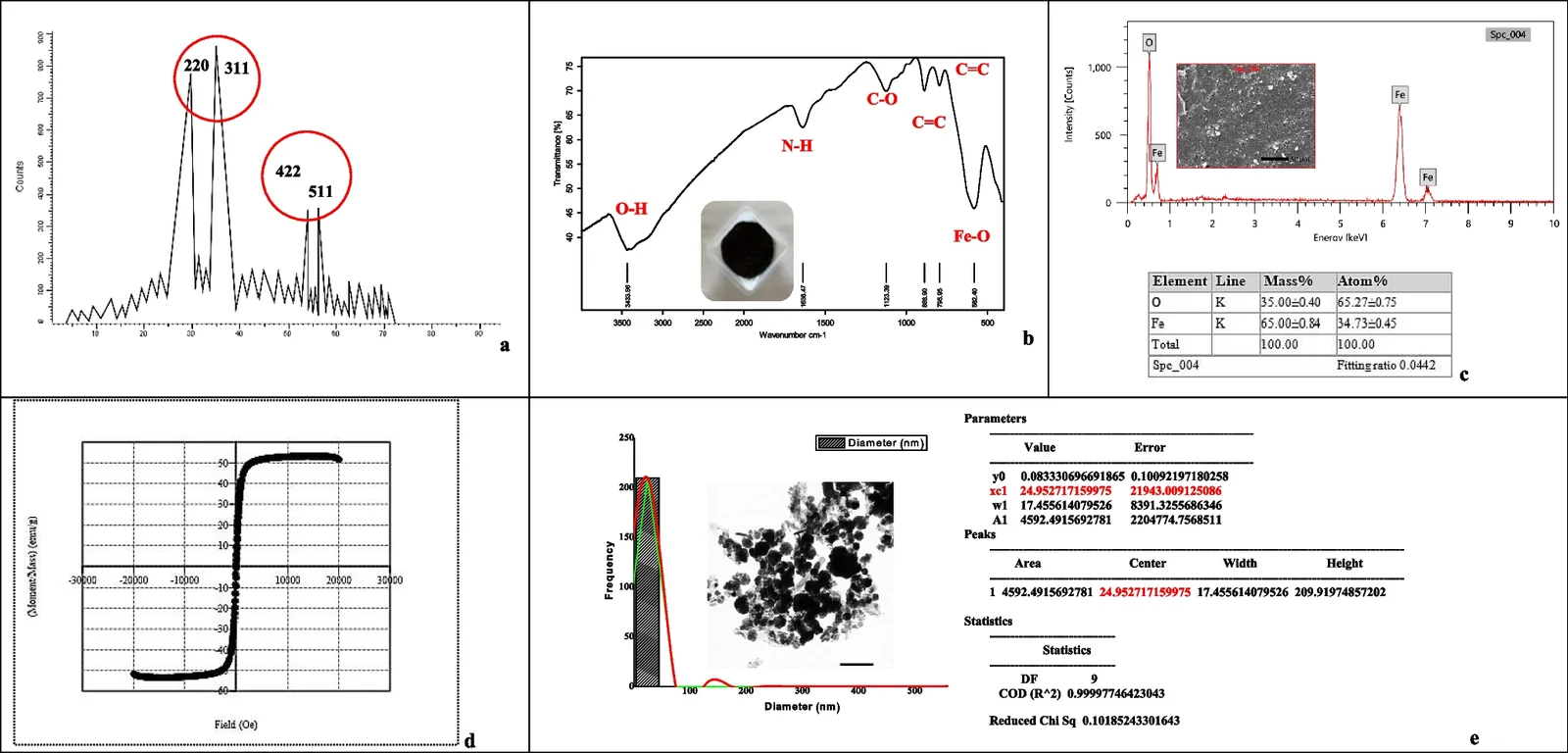
X-ray diffraction pattern of Fe_3_O_4_-SPIONs (**a**), FTIR spectrum of Fe_3_O_4_-SPIONs (**b**), SEM–EDX analysis of biosynthesized Fe_3_O_4_-SPIONs (**c**), VSM (vibrating sample magnetometer) of Fe_3_O_4_-SPIONs (**d**), TEM image of nFe_3_O_4_ shows the distribution of individual iron oxide nanoparticles have a size in the range of 1 ~ 80 nm with an average size of 24.9 nm (**e**)

The crystallinity of the produced nanoparticle is determined through the X-ray diffraction (XRD) approach to check the diameter of a crystallite. The structural analysis of the prepared sample (Fig. 3a) revealed four distinctive peaks at Bragg’s angle 2θ, with peak positions at 29.75°, 34.98°, 54.01°, and 56.08°. The crystallite dimensions determined using Scherrer Eq. (1) were 43, 29, 93, and 94 nm, accordingly, with a significant peak at 34.98° at 29 nm. These distinctive peaks for Fe_3_O_4_ nanoparticles correspond to the crystal lattice planes < 220 > , < 311 > , < 422 > , and < 511 > , correspondingly, that confirm the cubic spinel magnetite (Fe_3_O_4_) phase in structure. The FTIR wavenumber-related regions of the synthesized Fe_3_O_4_ were illustrated in Table 1, which indicates the corresponding functional groups and compound classes according to the IR Spectrum Table (Merck 2024).

**Table 1:** FTIR spectra and the peak positions of the major IR bands of Fe_3_O_4_-SPIONs

Standard peak wavenumber range cm^−1^	Peak wavenumber of Fe_3_O_4_ cm^−1^	Bond and compound class
3200 ~ 3600 cm^−1^	3433.9632	O–H stretching, strong, alcohol
1580 ~ 1650 cm^−1^	1636.4690	N–H bending, medium, amine
1087 ~ 1124 cm^−1^	1123.3882	C-O stretching, strong, sec. alcohol
885 ~ 895 cm^−1^	888.9014	C = C bending, strong, alkene
790 ~ 840 cm^−1^	795.9517	C = C bending, medium, alkene
500 ~ 600 cm^−1^	582.4008	Fe–O stretching, strong, magnetite

The overall result of FTIR analysis reveals that specific organic substances hold O–H, N–H, C-O, and C = C groups corresponding to alcohol, amine, secondary (sec.) alcohol, and strong/medium bands of alkene, respectively (Fig. 3b), that function as coatings in the produced iron oxide nanoparticles, resulting in their high dispersion and reducing their aggregation. SEM–EDX imaging confirmed the existence of elements such as Fe and O in Fe_3_O_4_-SPIONs (Fig. 3c). Henceforward, EDX proves that the synthesized nanoparticles are 100% pure iron oxide nanoparticles, whose elemental composition is summarized in Fig. 3c. In addition, the elemental composition for Fe and O reveals that the O/Fe (65.27/34.73) atomic ratio of the Fe_3_O_4_ examined was 1.879. Next, we measured the magnetization of our biofabricated magnetite (Fe_3_O_4_) at room temperature using a vibration sample magnetometer (VSM). We achieved the saturation magnetization (Ms) of the produced Fe_3_O_4_ nanoparticles to be 53.481 emu/g. The magnetization showed a much more closed hysteresis loop (Fig. 3d), resulting in small coercivity (Hci) (59.253 Oe) and remanence (5.3814 emu/g), indicating the superior performance of the superparamagnetic ability. A high-resolution 2-D image of the synthesized nanoparticle via transmission electron microscopy (TEM) enables the determination of its dimension and morphology. The Fe_3_O_4_-SPIONs have a spherical form and a mean size of 24.9 nm, according to the size distribution histogram (Fig. 3e). Moreover, the zeta-potential distributions of Fe_3_O_4_-SPIONs floating in water, as determined by dynamic light scattering, revealed a negative − 17.6 mV ζ-potential value, which confirms the good dispersion for the produced Fe_3_O_4_-SPIONs (data not shown).

### Developmental toxicity of magnetite SPIONs (Fe_3_O_4_-SPIONs)

In this experiment, eggs from red tilapia were used as an in vivo model to assess the biosafety of various concentrations of Fe_3_O_4_-SPIONs.

#### Egg retardation and hatchability rates (%)

In this study, fertilized eggs of red tilapia began to hatch during the 1st and 2nd days of the exposure time to Fe_3_O_4_-SPIONs. All eggs, both controlled and treated, were successfully hatched at a rate of more than 60%. The lowest hatching rate (50.0 ± 43.3%) was recorded in embryos exposed to 10 mg/l of Fe_3_O_4_-SPIONs (Table 2a). Retardation of egg development in the control (56.7 ± 49.3%), in comparison to the rest of the treated groups, was associated with saving the embryo from malformation and death by giving it sufficient time to develop while waiting for the completion of the exposure period. In contrast, adhesion and aggregation of Fe_3_O_4_-SPIONs on the fertilized eggs were the most notable reasons for collapsing the egg forward to death.

**Table 2:** Egg retardation and hatchability rates (%) ± SD on red tilapia embryo/larvae upon influenced to Fe_3_O_4_-SPIONs (**a**) and tenfold increases in concentrations of Fe_3_O_4_-SPIONs (**b**)

**a. Concentrations (mg/l)**	**Egg retardation (%)**		**Hatchability (%)**	
	**1st**	**2nd**	**1st**	**2nd**
0	56.7 ± 49.3	8.3 ± 10.4	3.3 ± 5.8	66.7 ± 16.1
5	13.3 ± 15.3	6.7 ± 2.9	68.3 ± 5.8	73.3 ± 10.4
10	25.0 ± 43.3	1.7 ± 2.9	15.0 ± 15.0	76.7 ± 15.3
15	28.3 ± 49.1	0.0 ± 0.0	8.3 ± 5.8	60.0 ± 52.0
30	00.0 ± 0.0	0.0 ± 0.0	16.7 ± 12.6	50.0 ± 43.3
**b. Concentrations (mg/l)**	**Egg retardation (%)**		**Hatchability (%)**	
	**1st**	**2nd**	**1st**	**2nd**
0	56.7 ± 49.3^a^	8.3 ± 10.4	3.3 ± 5.8^b^	66.7 ± 16.1
50	0.0 ± 0.0^b^	1.7 ± 2.9	10.0 ± 5.0^ab^	71.7 ± 15.3
100	0.0 ± 0.0^b^	3.3 ± 2.9	5.0 ± 5.0^b^	56.7 ± 41.6
150	68.3 ± 5.8^a^	3.3 ± 5.8	16.7 ± 2.9^a^	73.3 ± 10.4
300	81.7 ± 23.6^a^	0.0 ± 0.0	6.7 ± 5.8^b^	70.0 ± 22.9

Means in the same column with various superscripts are significantly different (*P* < 0.05). Values are means ± SD, 9 individuals per treatment

On the 1st day of exposure to tenfold increases in the experimental concentration of Fe_3_O_4_**-**SPIONs, significant (*P* < 0.05) variations were observed among the groups (Table 2b). The embryos and larvae exposed to 150 mg/l Fe_3_O_4_-SPIONs showed significantly (*P* < 0.05) the highest percentage of egg retardation and hatchability rates, reaching 68.3 ± 5.8% and 16.7 ± 2.9%, respectively. The hatchability rates and egg retardation on the second day showed nearly identical outcomes. Eggs exposed to 100 mg/l Fe_3_O_4_-SPIONs from all fertilized eggs were only 56.7 ± 41.6% able to hatch by the end of the exposure period, while in the rest of the groups, more than 65% of fertilized eggs were developed successfully to hatch yolk sac larvae.

#### Yolk sac diameter and stander length

Stander length (SL) and yolk sac diameter (YS) of red tilapia exposed to Fe_3_O_4_-SPIONs inclusion levels are shown in Table 3a, b. Yolk sac diameter was measured in four directions and presented as minimum and maximum values. The yolk sac diameter decreased gradually with increasing the concentration of Fe_3_O_4_-SPIONs, which may be caused by the navigational influence of the interaction between the yolk sac protein material and Fe_3_O_4_-SPIONs. However, the groups that were exposed to 10 mg/l of Fe_3_O_4_-SPIONs had significantly slower yolk sac absorption (*P* < 0.05), as shown by the largest maximum diameter of 1.16 ± 0.09 mm (Table 3a). The yolk sac’s absorbance follows almost the same trend in both the control and treatment groups. There were no noticeable changes even when the Fe_3_O_4_-SPIONs concentrations were increased by 10 times. Stander length during the 1st and 2nd days of exposure for newly hatched red tilapia larvae had the same trend in both control and treatment groups. However, on the 3rd day of exposure, the control groups recorded significantly the highest value of stander length (2.52 ± 0.19 mm) (Table 3b).

**Table 3:** Stander length and yolk sac diameter (mm) ± SD of red tilapia exposed to Fe_3_O_4_-SPIONs (**a**) and tenfolds increases in concentrations of Fe_3_O_4_-SPIONs (**b**)

**a. Concentrations (mg/l)**	**Yolk sac-diameter (mm)**			**Stander length (mm)**	
	**Min**	**Max**	**1st**	**2nd**	**3rd**
0	0.84 ± 0.10	1.07 ± 0.05^ab^	2.15 ± 0.38	2.38 ± 0.20	2.52 ± 0.19^a^
5	0.84 ± 0.05	1.09 ± 0.05^ab^	–	2.26 ± 0.16	1.98 ± 0.11^b^
10	0.84 ± 0.07	1.16 ± 0.09^a^	2.39 ± 0.10	2.41 ± 0.23	2.23 ± 0.21^ab^
15	0.86 ± 0.01	1.01 ± 0.03^b^	2.44 ± 0.04	2.16 ± 0.12	2.18 ± 0.09^b^
30	0.76 ± 0.06	1.00 ± 0.10^b^	2.36 ± 0.16	2.24 ± 0.22	*
**b. Concentrations (mg/l)**	**Yolk sac-diameter (mm)**			**Stander length (mm)**	
	**Min**	**Max**	**1st**	**2nd**	**3rd**
0	0.84 ± 0.10	1.07 ± 0.05	2.15 ± 0.38	2.38 ± 0.20	2.52 ± 0.19
50	0.79 ± 0.03	1.10 ± 0.08	2.34 ± 0.12	2.34 ± 0.04	*
100	0.90 ± 0.04	1.10 ± 0.18	2.47 ± 0.03	2.33 ± 0.04	2.42 ± 0.34
150	0.88 ± 0.10	1.16 ± 0.06	2.38 ± 0.07	2.20 ± 0.22	2.46 ± 0.13
300	0.87 ± 0.00	1.09 ± 0.06	2.55 ± 0.02	2.37 ± 0.08	2.50 ± 0.18

Means in the same column with various superscripts are significantly different ( *P* < 0.05). Values are means ± SD, 15 individuals per treatment, (–) no hatching, (*) all killed

On the other side, at tenfold increases in experimental concentrations, the rapid development noted in the embryonic stage and early hatching led to an increase in the SL in the treated groups on the 1st day of exposure. This advantage of increasing SL did not last so long that on the 2nd and 3rd days of exposure, the control group occupied the tallest SL in comparison with the groups exposed to Fe_3_O_4_-SPIONs (Table 3b).

#### Survival and malformation rates (%)

The survival and malformation of red tilapia embryos and larvae that were exposed to diverse amounts of Fe_3_O_4_-SPIONs were studied. As shown in Table 4a, 30 mg/l of Fe_3_O_4_-SPIONs exhibited the first toxicity signs in red tilapia embryos and larvae. After the 2nd day of exposure time, 30 mg/l of Fe_3_O_4_-SPIONs demonstrated toxicity, killing 50% of the red tilapia embryos and larvae. This result is linked to the retardation of egg development and malformation as distinguished in red tilapia embryos and larvae exposed to 30 mg/l of Fe_3_O_4_-SPIONs. By the end of the exposure time, the entire group had vanished. Moreover, on the 3rd day of the exposure time, the survival declined sharply to less than 50% at concentrations of 5 and 10 mg/l to 15.0 ± 26.0 and 30.0 ± 52.0%, respectively. This result recommends that the red tilapia development toxicity of Fe_3_O_4_-SPIONs is dose-dependent.

To learn more about how harmful Fe_3_O_4_-SPIONs are to cells, 10 times the experimental concentrations were also tested on red tilapia embryos and larvae (Table 4b). Contrary to what is expected, 10-folds of the experimental concentrations demonstrated considerable survival rates fluctuating around 70% to reach their lowest value (60.0 ± 39.7%) at embryos and larvae received to 100 mg/l of Fe_3_O_4_-SPIONs during the 2nd day of the exposure time.

**Table 4:** Survival and malformation rates (%) ± SD of red tilapia embryos and larvae exposed to Fe_3_O_4_-SPIONs (**a**) and 10-folds of Fe_3_O_4_-SPIONs concentrations (**b**) at specific time points

**a. Concentrations (mg/l)**	**Survival (%)**			**Malformation (%)**	
	**1st**	**2nd**	**3rd**	**2nd**	**3rd**
0	80.0 ± 10.0	75.0 ± 15.0	75.0 ± 15.0	0.0 ± 0.0	1.7 ± 2.9
5	86.7 ± 5.8	80.0 ± 10.0	15.0 ± 26.0	0.0 ± 0.0	0.0 ± 0.0
10	85.0 ± 17.3	78.3 ± 12.6	30.0 ± 52.0	0.0 ± 0.0	0.0 ± 0.0
15	81.7 ± 18.9	60.0 ± 52.0	58.3 ± 50.0	0.0 ± 0.0	0.0 ± 0.0
30	83.3 ± 7.6	50.0 ± 43.3	*	1.7 ± 2.9	*
**b. Concentrations (mg/l)**	**Survival (%)**			**Malformation (%)**	
	**1st**	**2nd**	**3rd**	**2nd**	**3rd**
0	80.0 ± 10.0	75.0 ± 15.0	75.0 ± 15.0^a^	0.0 ± 0.0^b^	1.7 ± 2.9
50	93.3 ± 7.6	75.0 ± 13.2	0.0 ± 0.0^b^	8.3 ± 7.6^a^	*
100	60.0 ± 52.2	60.0 ± 39.7	30.0 ± 52.0^ab^	0.0 ± 0.0^b^	0.0 ± 0.0
150	85.0 ± 5.0	76.7 ± 10.4	28.3 ± 49.1^ab^	1.7 ± 2.9^b^	1.7 ± 2.9
300	88.3 ± 20.0	70.0 ± 22.9	31.7 ± 54.8^ab^	0.0 ± 0.0^b^	0.0 ± 0.0

Means in the same column with various superscripts are significantly different (*P* < 0.05). Values are means ± SD, 9 individuals per treatment, (*) all killed

Some of the afflicted embryos were incapable of hatching and ultimately perished. However, on the 2nd day, malformation on newly hatched larvae was noted at concentrations of 50 and 150 mg/l. Because of this, the malformations were worse in the embryos and larvae given 50 mg/l treatment; 8.3 ± 7.6% of the embryos and larvae had significant (*P* < 0.05) spinal and craniofacial malformations and embryo retardation. In the 150 mg/l treatment group, the embryos and larvae showed malformation throughout the 1st, 2nd, and 3rd day exposure times, although there was no significant difference between the 100 and 300 mg/l treatment groups.

### Embryogenesis teratogenicity changes in red tilapia exposed to Fe_3_O_4_-SPIONs

Embryogenesis/larval malformation was divided in this study according to the morphological changes in the embryo and larval organization into two main categories: (1) egg coagulation and edema and (2) larval edema, spinal defects, and craniofacial malformation.

#### Egg coagulation and edema

**Fig. 4:**
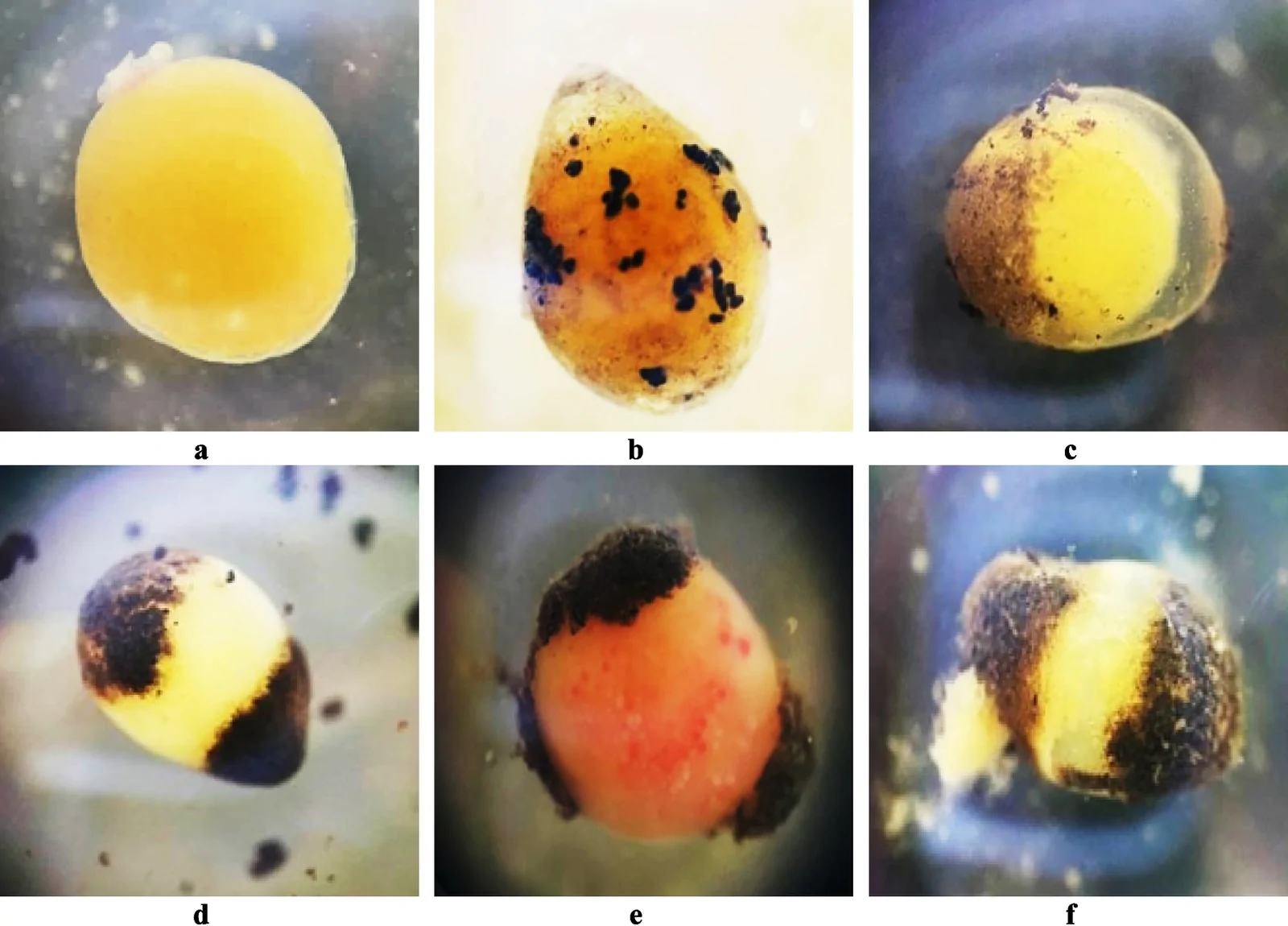
Fertilized egg malformations in red tilapia: fertilized egg control without any Fe_3_O_4_-SPIONs (**a**); the high accumulation load of Fe_3_O_4_-SPIONs (5 mg/l) on the surface of the egg membrane (**b**); egg coagulation and edema on the 1st day of exposure time with a concentration of 100 mg/l (magnification × 3.5) (**c**). Fertilized eggs showed malformations with Fe_3_O_4_-SPIONs clumped together on both sides of the egg pole. This causes the eggs to coagulate (50 mg/l) (**d**), coagulate with a bloody appearance (15 mg/l) (**e**), and collapse (15 mg/l) (**f**). (1st day of exposure time with magnification × 3.5)

Red tilapia eggs exposed to Fe_3_O_4_-SPIONs dispersed in embryonic media by straightforward soaking (Fig. 4b) adopted and absorbed nanoparticles via passive diffusion through the chorion pores as compared to the control (Fig. 4a). Due to the accumulation of Fe_3_O_4_-SPIONs, edema was noticed in the eggs. Furthermore, yolk mass in some eggs was coagulated toward the poles that were covered with the aggregated particles, creating a vacuum between the animal pole and yolk mass (Fig. 4c). In this study, the aggregation of the Fe_3_O_4_-SPIONs that covered both sides of the egg poles caused the whole egg material to coagulate and prevent cell division and proliferation (Fig. 4d). In some individual cases, blood spots were noticed on the coagulated eggs and invaded to turn them a bloody reddish color (Fig. 4e), which was possibly triggered by the crashing of the tiny blood vessels, as in hen eggs, when shocking or stressed (Patel et al. 2017). At late egg coagulation, the yolk material becomes degenerative, and the membrane ruptures, causing systematic egg death (Fig. 4f).

#### Larval edema, spinal defects, and craniofacial malformation

**Fig. 5:**
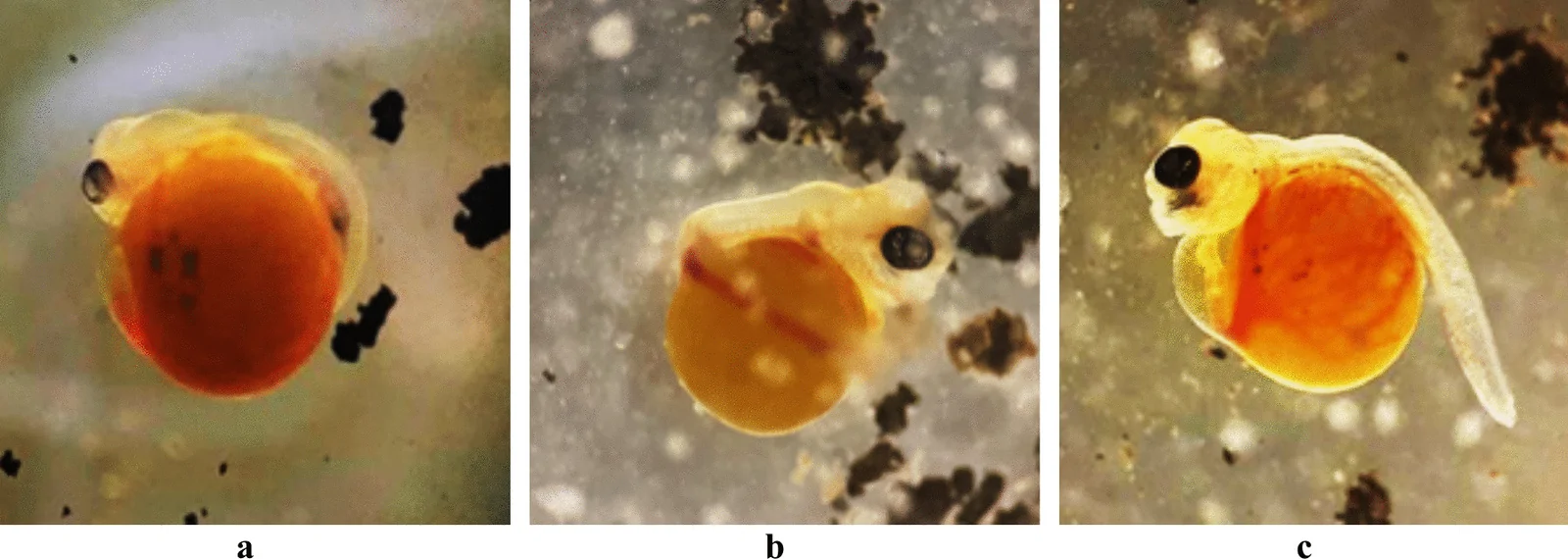
Yolk sac larval malformations in red tilapia showing yolk sac edema and curvature teratogenicity: scoliosis (lateral curvature) and early pericardial edema on the 1st day, 150 mg/l (**a**); scoliosis on the 2nd day, 30 mg/l (**b**); and kyphosis and late pericardial edema on the 2nd day, 50 mg/l (**c**)

The current observation clearly shows that the high concentration of Fe_3_O_4_-SPIONs on the red tilapia egg membrane caused swelling and serous fluid accumulation in the perivitelline space between the cell kernel and the membrane, initiating sublethal effects in all stages of development. In addition, pericardial edema, also referred to as yolk sac edema or yolk sac dropsy, is an increase in the volume of the pericardial cavity that results from the serous fluid assembling. In this study, two distinctive shapes of back malformation were identified. Figure 5a, b illustrates scoliosis, a condition where the larvae exhibit an abnormal lateral curvature of the spine. Also, kyphosis, an excessive outward curvature of the spine causing hunching of the back, was detected (Fig. 5c). It was also found that the red tilapia larvae had a C shape because the yolk sac absorbance was reduced, and the tail was curved sideways (Fig. 5a, b). Different groups were exposed to 150 mg/l (Fig. 5a) and 50 mg/l (Fig. 5c) of Fe_3_O_4_-SPIONs, which caused early and late pericardial edema.

**Fig. 6:**
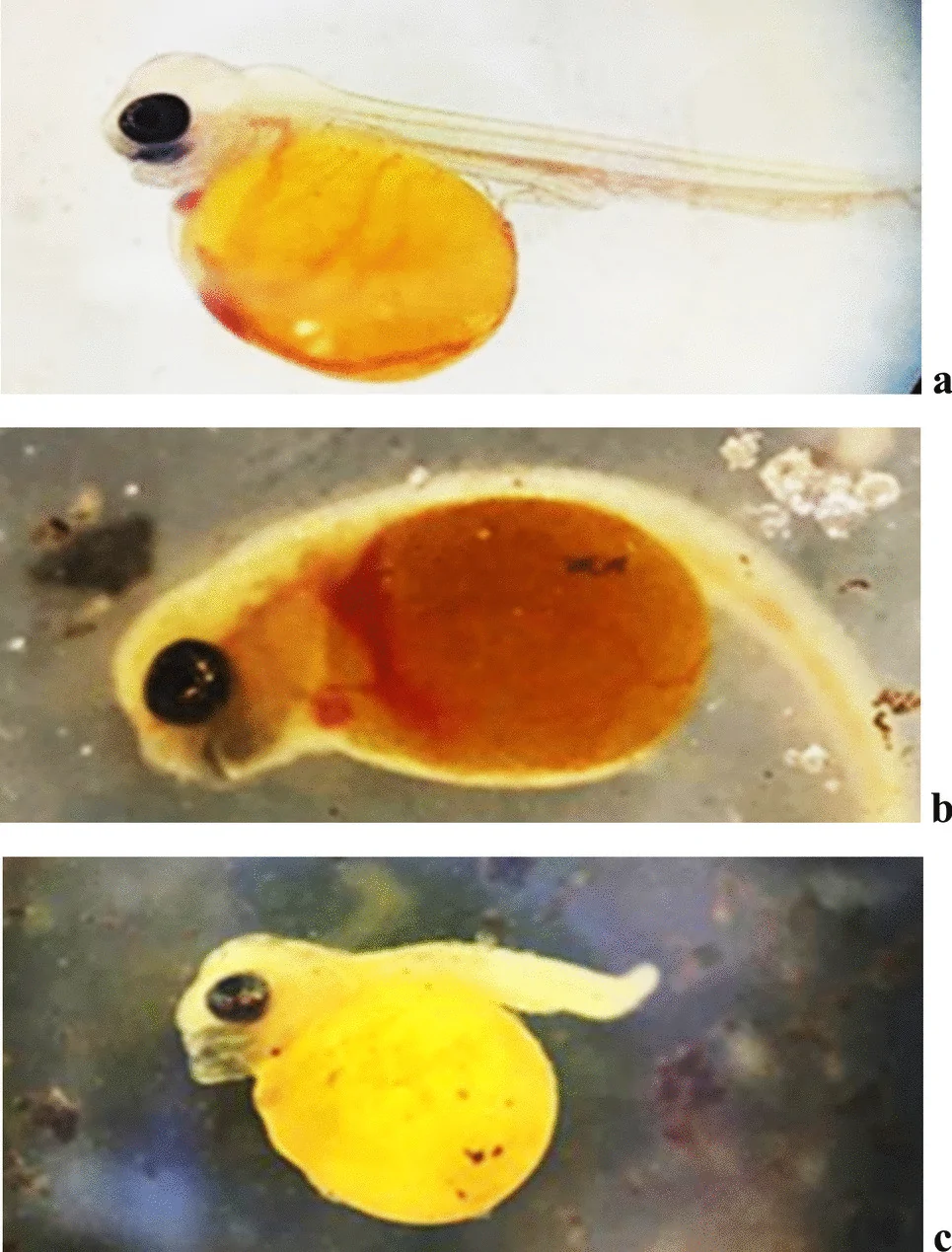
Lateral view of the normal red tilapia embryo (**a**); yolk sac larval malformations (curvatures teratogenicity) in red tilapia showing lordosis, axil curvature, lack of heart tube looping, and jaw deformation (Craniofacial) on the 2nd day, 50 mg/l (**b**); and craniofacial associated with larval stunting and late coagulation led to death on the 3rd day, 150 mg/l (**c**)

On the 3rd day after hatching, the skull of the control red tilapia was straightened out, and the lower jaw slowly lengthened until the mouth protruded. As the common cardinal vein came out around the yolk sac, blood flow started ventral to the floor plate of the spinal cord and the notochord in the caudal fin (Fig. 6a). The curvature in the back region of yolk sac larvae supplemented with Fe_3_O_4_-SPIONs was characterized by many forms. Craniofacial malformation was associated with spine malformation, whereas the jaw structure was deformed (Fig. 6b, c). Craniofacial was also associated with larval stunting and late coagulation that led to death (Fig. 6c).

No malformation was observed in red tilapia embryos or larvae receiving Fe_3_O_4_-SPIONs at concentrations of ≤ 10 mg/l. At 50 mg/l, however, the embryos and larvae developed severe malformations such as egg coagulation, lordosis, a lack of heart tube looping, and jaw deformation. As a result, some of the directly impacted embryos were unable to hatch and perished. Furthermore, by increasing the concentration to 150 mg/l, several forms of spinal defects were detected, including scoliosis and kyphosis. Also, craniofacial coagulation associated with stunting and late yolk sac larval coagulation was detected at 150 mg/l.

## Discussion

In this study, *Bacillus* sp. cells successfully produced superparamagnetic iron oxide nanoparticles of the magnetite type (Fe_3_O_4_-SPIONs) through biomineralization activity. The cells generated a dark brown-to-black colloid in response to a 50 mM FeSO_4_.7H_2_O challenge, indicating the development of SPIONs that responded quickly to external magnetic fields. As previously mentioned by Majeed et al. (2020), the color transformation serves as a visual and direct indication of nanoparticle formation. In the last few years, synthesizing and understanding SPIONs properties have increased significantly (Patil et al. 2018; Duli´nska-Litewka et al. 2019; Wu et al. 2015b; Samrot et al. 2021; Samrot et al. 2012). This is not the first study describing the biofabrication of SPIONs through bacteria. This fascinating material was extracellularly synthesized in a biologically induced biomineralization (BIM) manner as greigite (Fe_3_S_4_) when added with an iron source (Samrot et al. 2012) and by biologically controlled biomineralization (BCM) as mentioned previously by Revati and Pandey (2011). Sundaram et al. (2012) stated the capability of *Bacillus subtilis* strains isolated from rhizosphere soil to create extracellular Fe_3_O_4_ nanoparticles. During this study, the crystallinity of the generated nanoparticle was verified using X-ray diffraction (XRD). Four characteristic peaks at Bragg’s angle 2θ were observed, corresponding to the cubic spinel magnetite (Fe_3_O_4_) phase in the structure, with crystallite sizes calculated from the Scherrer equation. In accordance with the XRD analyzed data obtained from the produced Fe_3_O_4_-SPIONs, Singh et al. (2018) previously mentioned that magnetite possesses an inverse cubic spinel crystalline structure composed of Fe (III) ions that have been randomly assigned among octahedral and tetrahedral sites and show the reflection pattern as (220), (311), (400), (511), and (440). Sundaram et al. (2012) also studied the XRD peaks of the Fe_3_O_4_ and matched them with the existing ones in the Joint Committee on Powder Diffraction Standards (JCPDS). The characteristic peaks observed were at 2θ = 30.17°, 35.46°, 43.38°, 53.69°, 57.23°, and 62.77°, which corresponded to (220), (311), (400), (422), (511), and (440) Bragg reflection, respectively. This suggests that the manufactured Fe_3_O_4_ nanoparticles were cubic spinel in structure with an average dimension of about 70 nm. It is essential to improve it with biofunctional coatings to prevent the rapid oxidation of IONPs in oxygen-rich environments. This will improve their dispersion rate in water, prevent medicinal ingredients from decomposition, and have a substantial impact on their biokinetics and biodistribution in the body (Fernandes et al. 2017; Shen et al. 2018). According to this, we analyzed our magnetite SPIONs using FTIR to study their stability and functionalization. In accordance with our FTIR data, Sundaram et al. (2012) mentioned the FTIR spectra observed from the extracellularly manufactured iron oxide nanoparticles by the *Bacillus subtilis* strain as O–H, C = O, C-O, and N–H groups corresponding to specific organic compounds. The characteristic absorption peak that appeared in the IR spectrum of our magnetite at 582.4008 cm^−1^ is due to the Fe–O stretching vibration of Fe_3_O_4_. This observation was confirmed previously by Mohamed et al. (2017), who notified us of this particular absorption peak centered at 557 cm^−1^. Moreover, a high-resolution 2-D TEM image revealed a spherical shape with an average size of 24.9 nm for the biosynthesized (Fe_3_O_4_-SPIONs) nanoparticles. Samrot et al. (2021) created SPIONs of size about 25 nm that synchronized with the size obtained during this study, while Samrot et al. (2012) defined the superparamagnetic iron oxide nanoparticles as magnetite, maghemite, or any other ferrite with a dimension of no more than 100 nm and a superparamagnetic property, making the SPIONs an innovative nanoparticle for application in medical research as they can be controlled via an exterior magnetic field. In addition, Samrot et al. (2012) found that the dimension and morphology of the nanoparticle are critical to its characteristics. Using SEM–EDX analysis, this study showed that Fe and O elements were present in Fe_3_O_4_-SPIONs, confirming that the particles were 100% pure iron oxide with an O/Fe (65.27/34.73) atomic ratio of 1.879. In accordance with the EDX data obtained from the produced Fe_3_O_4_-SPIONs, Mohamed et al. (2017) studied the EDX pattern of Fe_3_O_4_ and detected its elemental composition as C (8.14 ± 0.14), O (54.66 ± 1.5), and Fe (37.20 ± 1.0). We also used a vibration sample magnetometer (VSM) to measure the biofabricated magnetite (Fe_3_O_4_-SPIONs) at room temperature. The VSM showed a closed hysteresis loop with low coercivity and remanence, which means the superparamagnetic properties were good, as shown by the magnetization measurement. Palanisamy and Wang (2019) previously demonstrated that particles become paramagnetic when their size is sufficiently small. Only in the existence of an applied magnetic field can they become highly magnetized when they are smaller than the critical size of 20 nm. However, magnetite nanoparticles, even in the size range of 35 nm, were described to show superparamagnetism (Samrot et al. 2021). Li et al. (2017) made magnetite nanoparticles (Fe_3_O_4_) with an Ms value of 54.7 emu/g. This is almost the same as the Ms value of the Fe_3_O_4_-SPIONs we produced. Furthermore, the ζ-potential value measured in water showed a negative − 17.6 mV for Fe_3_O_4_-SPIONs. The more negative ζ-potential value in the Fe_3_O_4_-SPIONs indicates the occurrence of hydroxyl groups at the nanoparticle’s surface. This fact implies that the electrostatic repulsion among the OH − groups on the nanoparticles’ surface accounts for the colloidal stability of the Fe_3_O_4_-SPIONs in water, as confirmed previously by Estévez et al. (2023).

In recent years, there has been a widespread application and intensive production of nanoparticles, leading to their release into the environment, particularly the aquatic ecosystem. The proliferation of nanomaterials in fish, leading to various pathological alterations in the host, increases fears. According to Savage et al. (2019), the increasing generation and extensive dispersion of nanoparticles could pose a considerable public health risk due to their catalytic activity and potential hazard to physiological processes. Additionally, studying the potential environmental effects of NP agglomerates, which settle out of the water column and become toxic, could be beneficial using red tilapia embryos (Nor et al. 2020; Huang et al. 2021). Previously, zebrafish embryos could study the developmental toxicity of magnetite SPIONs (Fe_3_O_4_-SPIONs). They can float on the surface of the water and sink to the bottom. This makes it possible for them to mimic the direct contact between organisms that live on the bottom of the water and nanoparticles in the sediment (Zhu et al. 2012). In this study, retardation in the development of red tilapia eggs in the control group kept embryos from being malformed or dying by giving them enough time to grow. On the other hand, Fe_3_O_4_-SPIONs stuck to and gathered on the fertilized eggs, which caused the eggs to fall apart. Cheng et al. (2007) suggested previously the cause of retarded embryo hatching and development upon adhering or adsorption of iron oxide NP agglomerates on the surface due to changes in surface physical features or intervention with the function of digestion of the chorionic hatching enzyme. In addition, it may result in exchanging oxygen depletion, leading to hypoxia in embryos upon contact. Early research has shown that doses of dimercaptosuccinic acid-coated iron oxide (Fe_3_O_4_) NPs greater than 50 mg/kg could hinder mouth embryonic growth, resulting in a substantial reduction in infant animal development (Noori et al. 2011). This study found that red tilapia eggs hatched at over 60% during exposure to Fe_3_O_4_-SPIONs, with the lowest rate in embryos exposed to 10 mg/l. In the same context, hatching delays and toxicity were also detected in zebrafish embryos subjected to ≥ 10 mg/l nFe_2_O_3_ (Zhu et al. 2012). The study revealed significant (*P* < 0.05) variations in egg retardation and hatchability rates among embryos and larvae exposed to different concentrations of Fe_3_O_4_-SPIONs. Those exposed to 150 mg/l showed the highest percentages, while those exposed to 100 mg/l had only 56.7 ± 41.6% hatchability. Despite these differences, over 65% of fertilized eggs successfully developed yolk sac larvae. Furthermore, Nations et al. (2011) demonstrated that, while nFe_2_O_3_ exposure (0.001 to 1000 mg/l) caused neither fatality nor substantial deformity in frog (*Xenopus laevis*) embryos and larvae, it did reduce tadpole SVL even at lower concentrations of 0.001 mg/l. Li et al. (2018) discovered that nano-ZnO interaction deformed *Mugilogobius chulae* embryos, leading to a significant decrease in the hatching rate. This study evaluated the stander length and yolk sac diameter of red tilapia exposed to varying amounts of Fe_3_O_4_-SPIONs inclusion. The diameter of the yolk sac decreased as the concentration of Fe_3_O_4_-SPIONs increased, potentially due to a reaction between the yolk sac’s protein content and the Fe_3_O_4_-SPIONs. On the other hand, those treated to 10 mg/l absorbed more slowly, with the maximal diameter measured at 1.16 ± 0.09 mm. Patel et al. (2017) hypothesized that proteins and nanomaterials interact, creating a “protein corona” and altering the biological identity of the former due to surface chemistry, size, and shape. In the same vein, Savage et al. (2019) clarified that the size, morphology, surface charge, coating, and chemical composition of nanoparticles can enhance their toxicity and response to biological systems, allowing them to penetrate the body’s defenses and deposit themselves in cytotoxic locations. Moreover, in chicken eggs, iron oxide nanoparticles interact with the egg albumen (Patel et al. 2017). The yolk sac absorbance and stander length of newly hatched red tilapia larvae showed similar trends in both the control and treatment groups. However, the control group recorded the highest stander length on the third day of exposure. At tenfold increases in concentrations, the embryonic stage and early hatching led to an increase in stander length in the treated groups. However, this advantage did not last long, and the control group occupied the tallest stander length compared to the treated groups. The impact of varying concentrations of Fe_3_O_4_-SPIONs on the survival and deformity of red tilapia embryos and larvae was investigated. At 30 mg/l, the first signs of contamination were noticed, and after 2 days, 50% of the population died. This has been connected to the deformity and retardation of egg development. By the end of the exposure period, the entire group had vanished. The survival rate dropped dramatically to fewer than 50% at 5 and 10 mg/l. Patel et al. (2017) conducted a study where they found that a dose range of 10–100 mg/ml of iron oxide nanoparticles (IONPs) could induce 50% mortality in chick embryos. This negative impact on embryonic development is thought to be due to changes in the molecular structure of albumen, which leads to alterations in the nourishing metabolism of the evolving embryo. Additionally, the study also tested 10 times the experimental concentrations of Fe_3_O_4_-SPIONs on red tilapia embryos and larvae. Despite the expected high survival rates, the concentrations showed significant malformations in newly hatched larvae. The worst malformations were observed in the 50 mg/l treatment, with 8.3 ± 7.6% of embryos and larvae showing significant spinal and craniofacial malformations and embryo retardation. The 150 mg/l treatment group also showed malformations throughout the exposure times, with no significant difference between the 100 and 300 mg/l treatment groups. The highest dose, 200 mg/ml or 3.32 mg IONPs/gm of egg, killed all the cells because of too many Fe^2+^ ions and complicated interactions between the ions and albumen and other biomolecules (Patel et al. 2017). It could, in particular, as previously reported by Singh et al. (2010) cause a disparity in homeostasis and abnormal cell-mediated reactions such as cellular toxic effects, destruction of DNA, oxidative strain, epigenetic modification, and inflammation, leading to the observed toxicity. This study categorized embryogenesis/larval malformation into three main categories: egg coagulation and edema, larval edema, spinal defects, and craniofacial malformation. Red tilapia eggs exposed to Fe_3_O_4_-SPIONs absorbed nanoparticles passively through chorion pores, causing edema and coagulation of yolk mass toward the covered poles. This aggregation may hinder cell division and proliferation, and in certain instances, blood spots may appear on the coagulated eggs, giving them a bloody reddish hue. At late egg coagulation, the yolk material becomes degenerative, and the membrane ruptures, causing systematic egg death. The study highlights the importance of understanding embryogenesis and larval malformations in animal health. Zhu et al. (2012) found that nFe_2_O_3_ aggregates (50 and 100 mg/l) harm zebrafish embryos and larvae, killing them in a dose-dependent manner, preventing hatching, and causing developmental defects including pericardial edema, deformation, and tissue ulcers. Some nanoparticles and their oxides, like iron oxides, have been shown to cause disease in common carp. This shows that they might have an effect on the liver and other parts of fish’s bodies, as shown by earlier studies (Tavabe et al. 2020; d’Amora et al. 2021; Alijantabar et al. 2022; Hajiyeva et al. 2024). These findings show that creatures in the preliminary phases of embryonic growth are more vulnerable to hazardous impacts. Studying living beings at these ages could thus aid in determining the sublethal consequences of nanoparticles and distinguishing between different kinds of toxic power (Hallare et al. 2004; Takeda et al. 2009; Zhu et al. 2012). A study by Hajiyeva et al. (2024) also discovered that Fe_3_O_4_ nanoparticles penetrated and bioaccumulated in the liver of juvenile common carp, causing significant damage to erythrocytes, hepatocytes, intracellular canaliculi, and bile ducts at concentrations of 10 and 100 mg. Savage et al. (2019) concluded that nanoparticles pose a threat to hosts through various mechanisms, including their physicochemical properties, which interact with proteins and cells. Catalyzing ROS generation contributes to their toxicological components, sparking oxidative stress and inflammatory signals, leading to necrosis, apoptosis, or carcinogenesis. Aggregation and sedimentation of metal nanoparticles on embryo/larval surfaces have been investigated in many studies as a main factor associated with embryo/larval collapse (Cheng et al. 2007; Zhu et al. 2012). He et al. (2011) proved in previous research that iron oxide nanoparticles on the surface of *Escherichia coli* damage the cell barrier and the exterior layer. Despite exposure to the same concentrations of iron nanoparticles, red tilapia embryos/larvae experienced high fluctuations during their development, particularly during hatching, which also affected survival rates and embryonic malformations. The observed aggregation process of iron nanoparticles during the experiment could potentially explain this. Savage et al. (2019) reported that both the size and form of nanoparticles have a major impact on how a substance moves through the body. Spherical nanoparticles undergo faster phagocytosis and secretion, while nanoparticles with a high aspect ratio have a four-fold increased uptake by HeLa cells. Furthermore, surface charge affects cellular uptake, with cationic and anionic charges increasing toxicity. Neutral surfaces are biocompatible, while zwitterionic particles are benign. Nanoparticles have the ability to disrupt lysosomal activity, breach skin barriers, and create “coronas” that masquerade as cationic particles. Furthermore, physical adhesion or adsorption may disrupt nutrition transfer among embryos and their surroundings. Nanomaterials can easily bind to different kinds of proteins, allowing for significant interactions in cultured cells (Ehrenberg et al. 2009) and organ systems (Chonn et al. 1992). As a result, they are recognized to get over biological barriers and effectively modify the physiological environment, which frequently results in toxic effects (Walkeyab and Chan 2012). Furthermore, the ability of plasma proteins to bind nanomaterials tends to be associated with harmful effects on cells (Clift et al. 2010). Nanomaterials interact with proteins in different manners, including establishing a “protein corona” (Lynch and Dawson 2008) and changing their “biological identity” owing to their dimensions, forms, and surface chemistry (Walkeyab and Chan 2012). Studies on chicken eggs discovered that iron oxide nanoparticles interact with egg albumen (Patel et al. 2017). It is hypothesized that interactions with egg albumen allow them to reach the developing embryo. In this study, high levels of Fe_3_O_4_-SPIONs cause the membranes of red tilapia eggs to swell and serous fluid to accumulate. These effects are not fatal at any stage of development. These results in pericardial edema, yolk sac edema, and two characteristic back malformations: scoliosis and kyphosis. The larvae’s curled tail and decreased yolk sac absorption contribute to their C-shaped appearance. Different concentrations of exposure result in early and late pericardial edema. To the best of our knowledge, spinal bending and edema are the most commonly observed and reported symptoms in marine animals exposed to nanoparticles (Li et al. 2018). Many investigations have demonstrated that iron oxide nanoparticles stick together and/or adsorb, which leads to excessive release of reactive oxygen species (ROS) like NO, O_2_, and N_2_ in living cells. This causes oxidative damage in the embryos and could possibly be a key factor in causing detrimental effects on development (Hassoun et al. 2005; Zhu et al. 2009). Also, Wehmas et al. (2015) show that zinc oxide nanoparticles are poisonous to zebrafish embryos and cause defects in the axial, craniofacial, somite, fin, and edema structures. These defects show that key processes during organogenesis are interrupted, or, in the case of edema, ion regulation is off. *M. chulae* embryos also went through a series of curvatures of the spine, hatching obstruction, skeleton abnormalities, and worsened vertebral bends when they were exposed to nano-ZnO (Li et al. 2018). As Choi et al. (2016) say, the pericardial and yolk sac edemata malformations that nano-ZnO may cause may be linked to changing immune system gene expression and inflammatory responses. There are also toxic substances that can cause spinal deformities, such as inflammation (Martens et al. 2012), protein kinase-like endoplasmic reticulum kinase (PERK) (Kupsco and Schlenk 2016), spinal bending (Cong et al. 2017), and ectopic expression of genes that control development (Cheng et al. 2000). Also, Mourad et al. (2022) illustrate the red tilapia larval distortion in the form of axial spine curvature in the caudal and abdominal regions. This study found that red tilapia larvae supplemented with Fe_3_O_4_-SPIONs developed malformations, including spine and jaw deformation, which led to stunting and late coagulation. No malformation was observed in embryos or larvae at concentrations of ≤ 10 mg/l. At 50 mg/l, embryos and larvae developed severe malformations, causing some to die. At 150 mg/l, spinal defects, scoliosis, and kyphosis were detected, along with craniofacial coagulation. This impact was also noticed in zebrafish embryos and larvae exposed to nFe_2_O_3_ at a concentration of 50 mg/l, which caused tissue ulceration, pericardial edema, and body arcuation (Zhu et al. 2012). Conclusively, the study investigates the environmental impact of toxic nanoparticles on red tilapia embryos. Results show that nanoparticle size, morphology, and coating can enhance nanoparticle toxicity. High concentrations of Fe_3_O_4_-SPIONs can lead to edema, yolk mass coagulation, spinal defects, and craniofacial malformations, highlighting the need to understand embryogenesis and larval malformations in animal health. Multiple techniques are required to understand a material’s toxicity profile, as biological systems can obscure causes, and there is no single gold standard for foreshadowing the toxicity of a unique nanomaterial. There is no universal method for revealing evidence about the toxicity or biocompatibility of nanoparticles in in vitro assays. We need multiple cell types and assays to replicate in vivo conditions. We also need to combine multiple assays to comprehend the physiological responses to nanoparticle systems. Future efforts focus on addressing these issues in nanoparticle toxicity analysis. This study is considered one of the leading and demonstrates how exposure to different amounts of Fe_3_O_4_-SPIONs alters the shape of red tilapia (*Oreochromis* sp.) embryos and larvae. This highlights the potential harm to aquatic organisms and ecosystem components. These results suggest that additional research is necessary to comprehend the vulnerability of aquatic species to the toxicity of Fe_3_O_4_-SPIONs and other ecologically benign nanoparticles.

## Data Availability

All data supporting the findings of this study are available within the paper and from the corresponding author on reasonable request.
