# Correction: Anxiety of microbially synthesized Fe3O4-SPIONs on embryonic/larval ontogeny in red tilapia (Oreochromis sp.)

**DOI:** 10.1007/s00253-025-13499-x

**Published:** 2025-06-09

**Authors:** Samia S. Abouelkheir, Mona M. Mourad

**Affiliations:** National Institute of Oceanography and Fisheries (NIOF), Cairo, Egypt

**Correction: Applied Microbiology and Biotechnology (2025) 109:3**

10.1007/s00253-024-13386-x

The history dates of this published article are incorrect.

The incorrect dates are:

Received: 26 November 2024

Revised: 26 November 2024

Accepted: 16 December 2024

And the correct dates should be:

Received: 04 April 2024

Revised: 26 November 2024

Accepted: 17 December 2024

The original article has been corrected.

